# Delusional parasitosis in dementia with Lewy bodies: a case report

**DOI:** 10.1186/s12991-019-0253-3

**Published:** 2019-12-30

**Authors:** Sho Ochiai, Hiroko Sugawara, Yusuke Kajio, Hibiki Tanaka, Tomohisa Ishikawa, Ryuji Fukuhara, Tadashi Jono, Mamoru Hashimoto

**Affiliations:** 1 Department of Neuropsychiatry, Kumamoto University Hospital, Kumamoto, Japan; 2 Yatsushiro Hospital, Yatsushiro, Japan; 3 Faculty of Social Welfare, Kumamoto Gakuen University, Kumamoto, Japan; 4 Department of Behavioral Neurology and Neuropsychiatry, Osaka University United Graduate School of Child Development, Osaka, Japan

**Keywords:** DLB, Delusional parasitosis, Aripiprazole, Donepezil

## Abstract

**Background:**

Dementia with Lewy bodies (DLB) is characterized by fluctuating cognitive impairments, recurrent visual hallucinations, the motor symptoms of parkinsonism and REM sleep behavior disorder. Various neuropsychiatric symptoms including hallucination and delusions occur frequently; however, delusional parasitosis is rare in DLB. Here, we report a case of DLB patient with delusional parasitosis.

**Case presentation:**

The patient was an 89-year-old woman. At the age of 88, she began to complain her oral cenesthopathy, and developed cognitive decline, delusional parasitosis and parkinsonism. As a result of examination, she was diagnosed as DLB and treated with combination of donepezil 5 mg/day and aripiprazole 1.5 mg/day, and her complaint was disappeared.

**Conclusions:**

Further studies are needed to investigate the association between delusional parasitosis and underlying pathophysiology of DLB, and the utility of antipsychotics for delusional parasitosis in DLB has to be examined through more cases.

## Background

Dementia with Lewy bodies (DLB) is recognized as the second common type of progressive neurodegenerative dementia in elderly people following Alzheimer’s dementia (AD) [1, 2], with core features characterized by fluctuating cognitive impairments, recurrent visual hallucinations, the motor symptoms of parkinsonism and REM sleep behavior disorder [3]. Various neuropsychiatric symptoms such as hallucinations and delusions occur more frequently in DLB than in AD patients [4], and among delusions, delusional misidentifications including Capgras syndrome associated with visual hallucinations represent the most frequent disturbance [5, 6].

Delusional parasitosis, which is characterized by a fixed and persistent belief of having a pathogenic infection despite objective evidence to the contrary, is more common in dermatology rather than psychiatry [7]. It presents the distinction between primary and secondary delusional parasitosis; the former cannot be explained by any other condition, the latter can result from psychiatric disorders, substances of abuse, medication, and general medical conditions including organic brain disease such as dementia [8]. Here, we report the case of a DLB patient with delusional parasitosis.

## Case presentation

The patient was an 89-year-old woman. She had educational background with high school graduation. She had no medical history except for hypertension, and no family history of neuropsychiatric illness. She had helped for her son’s home business; however, she gradually seemed to lose her energy with hearing loss and muscle weakness. She began to complain her oral cenesthopathy, which is a feeling of filament structures in her mouth, and visited another hospital at the age of 88. She developed progressive cognitive decline with decreasing Mini-Mental State Examination (MMSE) score from 26 to 22 in half of a year, and become depressive. She was treated with mirtazapine up to 22.5 mg/day for her depressive symptoms; however, her complaint had changed to delusional parasitosis, which is a belief of filaria infection in her nose and eyes, and her parkinsonism, such as resting tremor, muscle rigidity and bradykinesia, had developed. She admitted to our department for the purpose of examination and treatment for her cognitive impairment, delusion and parkinsonism. Her MMSE score was 22, which lost some points on orientation (− 1), serial-7 (− 3), three-stage command (− 1), delayed recall (− 2), and copy of double pentagon (− 1). The score of Geriatric depression scale was 8, and that of the United Parkinson’s Disease Rating Scale part 3 was 45. The results of blood test were normal, and an electroencephalogram was unremarkable. In her brain images, a brain magnetic resonance imaging scan showed mild atrophy of prefrontal cortex and bilateral hippocampus, and a single photon emission computed tomography showed reducing cerebral blood flow in bilateral thalamus, in addition to the parts of bilateral prefrontal, temporal, occipital lobe and parietal association area. Furthermore, 123iodine-metaidobenzylguandine myocardial uptake was reduced. Considering all the results of examinations, she was diagnosed as probable DLB. She was treated with donepezil up to 10 mg/day for a month; however, she continued to complain “Insects and snakes enter my body from my anus and deprive my nutrition”. For her depressive symptoms, aripiprazole augmentation with mirtazapine was performed, and her parasitosis delusion was disappeared keeping dose of mirtazapine 22.5 mg/day and aripiprazole 3 mg/day for a month. After that, she developed aspiration pneumonia, and her delusional parasitosis was exacerbated along with discontinuation of aripiprazole and mirtazapine. Finally, she was treated with a combination of donepezil 5 mg/day and aripiprazole 1.5 mg/day for a month, and her parasite delusion was disappeared.

## Discussion and conclusions

In the diagnosis, this case was difficult to differentiate between Parkinson disease with psychosis and depression and DLB. However, this case developed progressive cognitive decline before the onset of parkinsonism, and her cognitive impairment was similar to the characteristics of DLB, with greater deficits in attention and visual perception/construction and relatively preserved memory function [9]. Considering her clinical features and all the results of examinations, we diagnosed this case as having DLB.

Among the neuropsychiatric symptoms in DLB, visual hallucinations have been identified as one of the core clinical features in the criteria for the clinical diagnosis of DLB. On the other hand, systematized delusions have been identified as one of the supportive clinical features [3]. In our recent study, the frequency of delusion in DLB patients was 54.8% (68/124), top three of delusions were phantom boarder delusion, delusion of theft and persecution, and there was no case of delusional parasitosis [10]. Delusional parasitosis is rare in DLB, and there is only one case report [11]. Considering that organic brain disease including dementia is one of the potential aetiologies of secondary delusional parasitosis [8], delusional parasitosis could be present in DLB.

As well as systematized delusions, severe sensitivity to antipsychotic agents has been identified as one of the supportive clinical features in DLB [3]. In the previous report, quetiapine and olanzapine were successful for the treatment of DLB with delusional parasitosis [11]. Aripiprazole has a unique pharmacological profile acting as a dopamine D2 receptor partial agonist, which has been posited to reduce the risk of extrapyramidal symptoms [12]. However, the utility of aripiprazole for DLB has been conflicting in some previous reports [13–15]. Depressive symptoms can precede the onset of neurocognitive dysfunction in DLB [16], and Iwamoto et al. reported the effect of aripiprazole augmentation for depressive symptoms in the progression status of DLB [14]. In our case, she developed delusional parasitosis associated with depression, suggesting that aripiprazole might improve depressive symptoms including delusional parasitosis. In the previous reports, delusional parasitosis caused by dopamine agonists in patients with Parkinson disease [17, 18], and antipsychotics were effective in the majority of primary delusional parasitosis [19]. Finally, combination of donepezil and aripiprazole was effective for delusional parasitosis in our case of DLB. Recent preclinical findings point to the ability of antipsychotics to block the uptake of dopamine via dopamine transporter (DAT) [20], and the deriving increased extracellular dopamine from blocking the uptake has been suggested to have therapeutic effects especially on positive symptoms [21, 22]. Aripiprazole inhibits the locomotion of rodents stimulated by DAT blockers [23], and the effect we found in our case might be attributable by the ability of aripiprazole to increase extracellular dopamine and to block post-synaptic neurons of the indirect pathway via dopamine receptors. Furthermore, the effect of inhibition of acetylcholine release in the striatum by dopamine could be buffered by administering donepezil. Therefore, in our case, the increase in dopamine induced by aripiprazole improved delusional parasitosis via this alternative mechanism and the use of donepezil might have helped to increase acetylcholine signaling thus indirectly helping dopamine to be more effective. Further studies are needed to investigate the association between delusional parasitosis and pathophysiology of DLB, and the utility of antipsychotics for delusional parasitosis in DLB has to be examined through more cases.

## Data Availability

Not applicable.

## Abbreviations

DLB: dementia with Lewy bodies
AD: Alzheimer’s dementia
DAT: dopamine transporter
